# Prevalence of valvular heart diseases and associated risk factors in Han, Uygur and Kazak population in Xinjiang, China

**DOI:** 10.1371/journal.pone.0174490

**Published:** 2017-03-29

**Authors:** Yong-Tao Wang, Jing Tao, Ailifeire Maimaiti, Dilare Adi, Yi-Ning Yang, Xiao-Mei Li, Xiang Ma, Fen Liu, Bang-Dang Chen, Yi-Tong Ma

**Affiliations:** 1 Department of Cardiology, First Affiliated Hospital of Xinjiang Medical University, Urumqi, P. R. China; 2 Xinjiang Key Laboratory of Cardiovascular Disease Research, Urumqi, P. R. China; Osaka University Graduate School of Medicine, JAPAN

## Abstract

**Background:**

Valvular heart diseases (VHD) is very common in clinical practice and has became the subject of growing attention in the field of cardiovascular medicine. Our aim was to assess the prevalence and correlates of VHD in the general population in Xinjiang, China.

**Methods:**

Using a 4-stage stratified cluster random sampling method, a total of 14618 participants were recruited in the Cardiovascular Risk Survey (CRS) study. The participants’ personal information, medical history were assessed by questionnaire. VHD was diagnosed by transthoracic echocardiography. We carried out the statistical analysis utilizing SPSS Statistics version 19.0.

**Results:**

In the total study group, VHD was observed in 1397 (9.65%) individuals. The prevalence rates of VHD in Han, Uygur and Kazak group are 13.51%, 2.71% and 12.29% respectively. The prevalence rates of VHD increased strikingly with age (all P < 0.001). The results of multinomial regression analysis indicated that VHD were related to age in Han group, to age smoking and hypertension in Uygur group, to age and hypertension in Kazak group.

**Conclusion:**

Our research provides a unique prevalence rate of VHD in Xinjiang natural population. The result suggests that VHD are notably common in this population (9.65%) and increase with age. There exists significant difference of prevalence rate between ethnics. The main risk factors of VHD are age, hypertension and smoking. Valvular heart diseases should be regarded as a serious and growing public-health problem.

## Introduction

In the past two decades, the pattern of global disease has changed considerably, from primarily communicable, maternal, and perinatal causes to non-communicable disease (NCD) [1,2]. According to the statistics of World Health Organization in 2012, 17.6 million people died of Cardiovascular disease (CVD) worldwide, and proportionally, this accounts for 31.43% of global mortality [3]. CVD has become the most largest and important cause of NCD deaths. Valvular heart diseases (VHD), as a common disease in clinical practices, is an important cause of increased morbidity and mortality [4–9]. The national prevalence of VHD in adults older than 18 years in the US is 2.5% [10]. The result of this study also show that prevalence of VHD increased with age, from 0.7% in 18–44 years old to 13.3% in the 75 years and older group. Lu et al. performed a cross-sectional study and found that the total prevalence of VHD was 5.3% in Hunan Shaoyang area in China. The distribution of prevalence in ages was:1.5% between 18 and 29 years old, 2.7% between 30 and 39 years old, 1.8% between 40 and 49 years old, 4.8% between 50 and 59 years old, 13.2% between 60 and 74 years old [11]. The prevalence increased substantially with age too. The strong association between VHD and age, combined with the aging Chinese population, means that VHD has been described as the ‘next cardiac epidemic’ in China. The role of VHD as a severe public-health issue should be reconsidered. While the prevalence of VHD in China remains unclear.

Xinjiang is a multi-ethnic co-populated area. Among the 13 native ethnicities, the Uygur people account for 46%, and the Han account for 40%. We noted that different ethnic groups in Xinjiang may have different genetic backgrounds, diet and living environment which are risk factors of VHD[12,13]. However, limited information is available about the current burden status of VHD at the regional population level in Xinjiang province. Therefore, to evaluate the current status of VHD burden, as well as to treat earlier, this study provided an up-to-date assessment of VHD prevalence in a large population involving 14618 residents through a cross-sectional epidemiological survey, and investigated correlates of VHD in different ethnic in the region.

## Method

### Ethics statement

This study was approved by the Ethics Committee of the First Affiliated Hospital of Xinjiang Medical University and was conducted according to the standards of the Declaration of Helsinki. Written, informed consent was obtained from the participants.

### Study population

All the participants were selected from the Cardiovascular Risk Survey (CRS) study which is a multi-ethnic, community-based, cross-sectional study designed to investigate the prevalence, incidence and risk factors for cardiovascular diseases [12,14–15]. The CRS study was conducted from October 2007 to March 2010 in seven geographically distinct sites in Xinjiang Province, China: Urumqi City, Kelamayi City, Hetian Prefecture, Zhaosu City, Fukang City, Tulufan Prefecture, and Fuhai City. Briefly, a 4-stage stratified cluster random sampling was used to recruit residents from Xinjiang, northwest of China. The first level of stratification sampling involved cities. Based on population, ethnicity, geography, economic and cultural development level, the seven area mentioned above were selected in this study. Based on the ethnic aggregation status, one district or county was randomly selected from the Uygur population dominated area. The third level of stratifcation was the random selection of community or town (village) from each district or county. The fourth level of stratifcation was to select sujects aged above 35 years from each community or town (village) as research subjects. The staff conducted surveys in each household and administered questionnaires. Information regarding the demographics, socioeconomic status, dietary habits, and the medical history of each participant was collected. The CRS study included 14,618 participants who had been recruited in 2007–2010. Our study population included 14478 individuals from the CRS study who were Han, Uygur and Kazak.

### Specimen collection and laboratory testing

The participants were interviewed, had a physical examination, and blood drawn for laboratory testing. Blood samples were collected in vacutainer tubes containing EDTA from an antecubital vein in the morning after an overnight fasting period. Blood samples were centrifuged within two hours at the survey site and all the samples were transported on dry ice at prearranged intervals to Xinjiang coronary artery disease VIP laboratory. The serum concentration of serum total cholesterol, triglyceride, low density lipoprotein (LDL), high density lipoprotein (HDL) and fasting glucose were measured by the Clinical Laboratory Department of the First Affiliated Hospital of Xinjiang Medical University.

Risk factors for VHD included hypertension, diabetes, dyslipidemia and smoking. Hypertension was defined as self-reported use of antihypertensive medication within the past 2 weeks or an average systolic blood pressure 140 mmHg, an average diastolic blood pressure 90 mmHg, or both. Diabetes was present if each individual was either diagnosed by physician or had a fasting blood glucose of at least 7.0 mmol/L. Dyslipidaemia was defned as present if the individual had any one of the following conditions: (1) known case of dyslipidemia, or (2) one of these abnormal lipid profles, which were total cholesterol of at least 6.22 mmol/L, triglyceride concentration of at least 2.26 mmol/L, LDL cholesterol concentration of at least 4.14 mmol/L or HDL cholesterol concentration bellow 1.04 mmol/L.

Simons ACUSON Cypress portable color Doppler ultrasound systems were used for echocardiography and interpreting the images and the valvular assessment included the structure and function of heart valve, valve thickening, echogenicity, calcification and flow situation. The severity of valvular stenosis was depended on the valve area and the mean pressure gradient across the restrictive orifce [16]. In addition, the severity of valvular regurgitation was based on a qualitative scale and classifed as mild (grade 1), moderate (grade 2) and severe (grades 3–4), according to the current ACC/AHA guidelines for the management of individuals with valvular heart disease [17]. In our study, we found little people exist tricuspid valve disease, so signifcant valvular diseases were defined as any mitral or aortic stenosis severity, moderate or severe aortic regurgitation and moderate or severe mitral regurgitation.

### Statistical analysis

The data analysis was performed using SPSS version 19.0 for Windows (SPSS Inc., Chicago, IL, USA). The measurement data are shown as the means±SD, and the differences between case and control subjects were assessed using an independent-sample t-test. Differences in the enumeration data, such as the frequencies of smoking, hypertension, diabetes and dyslipidemia between case and control subjects were analyzed using the chi-square test. Additionally, logistic regression analysis with effect ratios (odds ratio [OR] and 95% CI) were used to examine correlates of VHD. A P value < 0.05 was considered to be statistically significant.

## Results

From October 2007 to March 2010, a total of 14618 participants were enrolled in the cross-sectional study. The actual number of completed investigations was 14,478. Table 1 shows the basic characteristics of participants with and without VHD in different ethnics. Among the Han subjects, participants with and without VHD differed significantly with regards to age, prevalence of male, smoking, hypertension and diabetes (all P<0.05). Among the Uygur subjects, age and prevalence of male, smoking and hypertension were significantly higher in participants with VHD than in the participants without VHD (all P<0.05). We found that participants with VHD had higher age and prevalence of hypertension and lower prevalence of smoking compared with participants without VHD (all P<0.05). The general characteristics of participants were also presented in S1 Table in detail.

**Table 1:** Basic characteristics of participants with and without VHD in different ethnic.

Variable	Han			Uygur			Kazak		
	Control	Case	P	Control	Case	P	Control	Case	P
	(n = 4902)	(n = 766)		(n = 588)	(n = 128)		(n = 359)	(n = 503)	
**Age**	50.58±11.94	63.52±11.52	<0.001	50.29±12.83	61.84±12.71	<0.001	47.84±11.38	54.24±12.30	<0.001
**Men**	2412(49.2)	346(45.2)	0.038	1944(42.4)	74(57.8)	<0.001	1764(49.1)	235(46.7)	0.313
**BMI**	25.10±3.48	25.17±3.38	0.629	25.70±4.38	25.77±4.27	0.853	26.45±4.73	26.74±4.52	0.198
**Smoking**	1593(32.5)	201(26.2)	0.001	851(18.5)	41(32.0)	<0.001	1315(36.6)	146(290	0.001
**Hypertens**	1794(36.6)	424(55.4)	<0.001	1459(31.8)	77(60.2)	<0.001	1653(46)	379(75.3)	<0.001
**Diabetes**	360(7.3)	82(10.7)	0.001	264(5.8)	9(7.0)	0.542	132(3.7)	18(3.6)	0.913
**Dyslipide**	2588(52.8)	388(50.7)	0.27	2317(50.5)	63(49.2)	0.775	1582(44.1)	227(45.1)	0.649

**Fig 1:**
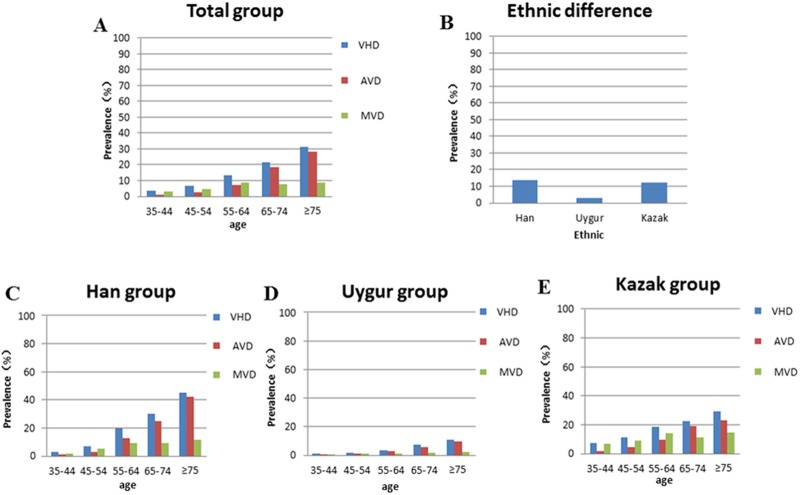
Prevalence of VHD across ethnic and age groups.

Prevalence of VHD on transthoracic echocardiography across ethnic and age groups are shown in Table 2 and Fig 1. In the total study population aged 35–101 years, VHD was observed in 1397 (9.65%) individuals, aortic valve disease(AVD) was observed in 841 (5.81%) individuals and mitral valve disease(MVD) was observed in 769 (5.31%) individuals. Prevalence rates of VHD, AVD and MVD increased strikingly with age (all P < 0.001). The prevalence rates of VHD in different age groups are 3.63%, 6.53%, 13.20%, 21.6% and 31.26% respectively. The prevalence rates of AVD in different age groups are 0.98%, 2.62%, 6.86%, 18.09% and 28.26% respectively and the prevalence rates of MVD in different age groups are 3.00%, 4.68%, 8.48%, 7.34% and 8.82% respectively. Among the participants ≤ 65 years, the prevalence rates of MVD is higher than AVD (4.84% VS. 2.91%, P < 0.001), while among the participants ≥ 65 years, the prevalence rates of AVD is higher than MVD (20.17% VS. 7.64%, P < 0.001).

**Table 2:** Prevalence of VHD across ethnic and age group.

	Age(years)					Total
	35–44	45–54	55–64	65–74	≥75	
**Total**						
**VHD**	196(3.63)	244(6.53)	383(13.20)	418(21.60)	156(31.26)	1397(9.65)
**AVD**	53(0.98)	98(2.62)	199(6.86)	350(18.09)	141(28.26)	841(5.81)
**MVD**	162(3.00)	175(4.68)	246(8.48)	142(7.34)	44(8.82)	769(5.31)
**Han**						
**VHD**	57(2.82)	96(7.08)	209(19.64)	290(29.84)	114(45.24)	766(13.51)
**AVD**	21(1.04)	36(2.65)	132(12.41)	243(25)	106(42.06)	538(9.49)
**MVD**	38(1.88)	66(4.87)	99(9.30)	89(9.16)	29(11.51)	321(5.66)
**Uygur**						
**VHD**	14(0.85)	22(1.76)	33(3.07)	41(7.13)	18(10.91)	128(2.71)
**AVD**	8(0.48)	15(1.20)	26(2.42)	33(5.74)	16(9.70)	98(2.08)
**MVD**	10(0.61)	11(0.88)	9(0.84)	9(1.57)	3(1.82)	42(0.89)
**Kazak**						
**VHD**	125(7.23)	126(11.14)	141(18.48)	87(22.42)	24(29.27)	503(12.29)
**AVD**	24(1.39)	47(4.16)	74(9.70)	74(19.07)	19(23.17)	238(5.81)
**MVD**	114(6.59)	98(8.66)	105(13.76)	44(11.34)	12(14.63)	373(9.11)

Abbreviations: VHD, valvular heart diseases; AVD, aortic valve disease; MVD, mitral valve disease

The prevalence rates of VHD in Han, Uygur and kazak group are 13.51%, 2.71% and 12.29% respectively. There exists significant difference between the three ethnics (P < 0.001) and the prevalence rates of VHD in Uygur group is significantly lower than the other ethnics. Markedly increased prevalence of VHD, AVD and MVD was observed with increasing age in the Han, Uygur and Kazak group respectively. (P all < 0.001). The prevalence rates of AVD and MVD in Han group are 9.49% and 5.66% respectively; The prevalence rates of AVD and MVD in Uygur group are 2.08% and 0.89% respectively. In the Han and Uygur group, the prevalence rates of AVD was significantly higher than MVD (P all < 0.001). While the prevalence rates of AVD and MVD in Kazak group are 5.81% and 9.11% respectively. In the Kazak group, the prevalence rates of AVD was significantly lower than MVD (P < 0.001).

Tables 3–5 show the multivariable logistic regression analyses of the major confounding factors for VHD. The multinomial variable was recoded into three categories: VHD, AVD and MVD. The independent variables were age, male, smoking, hypertension, diabetes and dyslipidemia. In the Han subjects, we found that older age was strongly associated with VHD, AVD and MVD. Besides, male and hypertension was signifcantly associated with MVD and AVD respectively. In the Uygur subjects, older age, smoking and hypertension were strongly associated with VHD and AVD. While, hypertension was significantly associated with MVD. In the Kazak, we found that older age and hypertension were both strongly associated with VHD, AVD and MVD.

**Table 3:** Multivariable logistic regression analyses in Han group.

	β	OR	95% CI of OR	P
**VHD**				
**age**	0.08	1.085	1.077–1.093	<0.001
**MVD**				
**age**	0.052	1.053	1.043–1.063	<0.001
**male**	0.395	1.485	1.101–2.001	0.009
**AVD**				
**age**	0.107	1.113	1.103–1.124	<0.001
**Hypertens**	0.207	1.23	1.003–1.508	0.047

Abbreviations: VHD, valvular heart diseases; AVD, aortic valve disease; MVD, mitral valve disease.

**Table 4:** Multivariable logistic regression analyses in Uygur group.

	β	OR	95% CI of OR	P
**VHD**				
**age**	0.06	1.062	1.046–1.077	<0.001
**Smoking**	0.615	1.85	1.157–2.956	0.01
**Hypertens**	0.742	2.1	1.445–3.053	<0.001
**MVD**				
**Hypertens**	1.085	2.881	1.493–5.561	0.002
**AVD**				
**age**	0.067	1.069	1.052–1.087	<0.001
**Smoking**	0.695	2.003	1.204–3.333	0.007
**Hypertens**	0.664	1.943	1.271–2.969	0.002

Abbreviations: VHD, valvular heart diseases; AVD, aortic valve disease; MVD, mitral valve disease.

**Table 5:** Multivariable logistic regression analyses in Kazak group.

	β	OR	95% CI of OR	P
**VHD**				
**age**	0.031	1.032	1.023–1.041	<0.001
**Hypertens**	1.006	2.733	2.177–3.432	<0.001
**MVD**				
**age**	0.016	1.016	1.006–1.026	0.001
**Hypertens**	1.015	2.761	2.139–3.563	<0.001
**AVD**				
**age**	0.073	1.075	1.062–1.089	<0.001
**Hypertens**	0.995	2.706	1.907–3.839	<0.001

Abbreviations: VHD, valvular heart diseases; AVD, aortic valve disease; MVD, mitral valve disease.

## Discussions

This survey is the first cross-sectional epidemiological study to assess the prevalence of VHD in Xinjiang, China. In our study, the prevalence rate of VHD was 9.65%. The prevalence rates of VHD in the current study were higher than those reported in previous studies. The discrepancies in prevalence may be accounted for the reasons as follow: first, the study population is different and there may be a racial difference in the prevalence of VHD [18]; Second, the health conditions between industrialized and developing countries are different [19]; Third, each study may adopt different definitions of VHD [20–21]. In our study, VHD contained symptomatic and asymptomatic VHD, while in other studies, the definition of VHD may be only symptomatic significant VHD.

Previous studies demonstrated that the prevalence of VHD increased markly with age [10,11]. Our research has also got the same results. The prevalence rates of VHD in different age groups (35–44 years, 45–54 years, 55–64 years, 65-74years and > 75 years) were 3.63%, 6.53%, 13.20%, 21.6% and 31.26% respectively. In all participants with valvular heart disease, AVD and MVD share the same prevalence rate (5.81% Vs. 5.31%, P >0.05). While, in Han and Uygur population, AVD is the most common etiology, and in Kazak population, MVD is the most common etiology. The reason of this discrepancy may be that the prevalence of dyslipidemia in Kazak population is significantly lower than that in Han and Uygur population. And high blood lipids level is a wellknown risk factor for calcific aortic valve disease. Thus the prevalence of AVD in Kazak population is low.

However, our study also showed that there exists racial difference in the prevalence of VHD. The prevalence rates of VHD in Han, Uygur and kazak population were 13.51%, 2.71% and 12.29% respectively. The prevalence rates of VHD in Uygur was significantly lower than that in Han and Kazak population. Prior studies have demonstrated that the pathology of calcific aortic valve disease involve chronic inflammation, lipids deposition, and calcium metabolism, all of which may differ between race-ethnic groups [22,23]. Yukiko Sashida performed a large multi-ethnic population-based cohort study to evaluate whether aortic valve thickness differs from race-ethnicity. 2085 participants including Hispanic (57%), non-Hispanic black (22%), and non-Hispanic white (21%) were enrolled. They found that there exists ethnic differences in the degree of aortic valve thickness. Hispanic ethnicity was a independently protective factor against aortic valve thickness [24]. Further, Devin K et al performed a study which contained a database of over 2.1 million patient records to identify the association of race with severe aortic stenosis in America. They found that African Americans are at obviously lower risk of developing severe aortic stenosis than Caucasians [25]. While the reason for this discrepancy in our study is still unclear and it may be a result of diet, genetic factors and environmental factors. Further genetic and laboratory investigation is warranted to determine the underlying mechanism for the lower prevalence of Uygur population.

Whether gender is a risk factor of VHD remains unclear. On one hand, Podolec et al. reported that female sex is responsible for degenerative valvular heart disease [26]. Boon A also reported that female sex was strongly and significantly associated with stenotic aortic valve calcification [27]. On the other hand, the Cardiovascular Health Study by Novaro GM suggested that male gender was related to risk of incident aortic stenosis [28]. Ferreira performed a cross-sectional study in a random sample of 1068 participants >65 years in a Mediterranean area and found that there exists no significant difference in the prevalence of calcific aortic valve disease between male and female [22]. In the present study, male gender is an independent risk factor of MVD in Han population and no association is found in other ethnic populations. The reason of this discrepancy may be caused by sampling error.

Many studies have demonstrated that smoking is significantly associated with VHD [29]. The role of smoking in the occurrence of VHD may be that smoking may adverse the permeability of endothelial and lipoprotein oxidation[30,31]. In the present study, we found significant association between smoking and VHD and AVD only in Uygur population. This may be explained by the fact that the prevalence of smoking in Han and Kazak were significantly higher than that in Uygur. Previous studies reported that 20% - 50% of senile degenerated VHD patients were diagnosed with hypertension [32]. In the present study, the prevalence of hypertension is significantly higher in VHD population than in controls in Han, Uygur and Kazak group. After multivariable logistic regression analysis, hypertension is still an independent risk factors of VHD.

The present study has several limitations. First, it was a cross-sectional study and we failed to get a cause-and-effect relationship between risk factors and VHD. Second, the participants might have the recall bias, which may result in the difference in information provided. Third, for the reason of economical and human resource constraints, data including coronary artery disease and rheumatic fever were lacking.

## Conclusion

In conclusion, our research provides a unique prevalence rate and distribution of VHD in Xinjiang natural population. Through the epidemiological investigation, we found that valvular heart diseases were notably common in Xinjiang and increase with age. The prevalence of VHD in Xinjiang is higher than that in other area in China. VHD is a asymptomatic and chronic disease with long incubation period and echocardiography is the only method to diagnose. Most patients with VHD are diagnosed after symptoms and not all elderly patients can be candidates for surgery. Increasing number of people have gradually realized the high prevalence and serious consequences of VHD, thus it should be regarded as a serious and growing public-health problem.

## Supporting information

S1 Appendix Survey questionnaire. (DOCX)

S2 Appendix STROBE checklist. (DOCX)

S1 Table Supporting table. (DOCX)
