# Supplementary material for: Prevalence of valvular heart diseases and associated risk factors in Han, Uygur and Kazak population in Xinjiang, China
Source: PLoS One. 2017 Mar 29;12(3):e0174490. doi: 10.1371/journal.pone.0174490 (PMC5371360; doi:10.1371/journal.pone.0174490)
Supplement: S1 Appendix — (DOCX) [file pone.0174490.s001.docx]

| 样本编号 |  | 姓名 |  | 性别 |  |
| --- | --- | --- | --- | --- | --- |
| 出生年月 |  | 族别 |  | 职业 |  |
| 受教育程度 |  | 婚姻状况 |  | 联系电话 |  |
| 身高 |  | 体重 |  | 血压 |  |
| 吸烟史 |  | | | | |
| 饮酒史 |  | | | | |
| 绝经史 |  | | | | |
| 住址 |  | | | | |
| 个人病史及用药情况 |  | 病程 | | 服药史 | |
|  | 冠心病病史 |  | |  | |
|  | 高血压病史 |  | |  | |
|  | 高血脂 |  | |  | |
|  | 心律失常 |  | |  | |
|  | 心力衰竭 |  | |  | |
|  | 先天性心脏病 |  | |  | |
|  | 瓣膜病 |  | |  | |
|  | 糖尿病病史 |  | |  | |
| 家族史 | 父亲 |  | | | |
|  | 母亲 |  | | | |
|  | 兄弟姐妹 |  | | | |

| Sample number |  | Name |  | Gender |  |
| --- | --- | --- | --- | --- | --- |
| Date of birth |  | Ethnic |  | Occupation |  |
| Education level |  | Marital status |  | Phone number |  |
| Height |  | Weight |  | Blood pressure |  |
| Smoking history |  | | | | |
| Drinking history |  | | | | |
| Menopause history |  | | | | |
| Address |  | | | | |
| Personal history  and Drug use |  | Course of disease | | Drug use | |
|  | Coronary heart disease |  | |  | |
|  | Hypertension |  | |  | |
|  | Hyperlipemia |  | |  | |
|  | Arrhythmia |  | |  | |
|  | Heart failure |  | |  | |
|  | Congenital heart disease |  | |  | |
|  | Valvular heart disease |  | |  | |
|  | Diabetes |  | |  | |
| Family history | Father |  | | | |
|  | Mother |  | | | |
|  | Brothers and sisters |  | | | |
