# Supplementary material for: Prevalence of valvular heart diseases and associated risk factors in Han, Uygur and Kazak population in Xinjiang, China
Source: PLoS One. 2017 Mar 29;12(3):e0174490. doi: 10.1371/journal.pone.0174490 (PMC5371360; doi:10.1371/journal.pone.0174490)
Supplement: S1 Table — (DOCX) [file pone.0174490.s003.docx]

S1 Table

|  | Control（n=13081） | VHD(n=1397) | P |
| --- | --- | --- | --- |
| Age | 49.73±12.17 | 60.03±12.69 | ＜0.001 |
| Men | 6120(46.8%) | 655(46.9%) | 0.943 |
| BMI | 25.68±4.21 | 25.79±3.98 | 0.36 |
| Ethnic |  |  | ＜0.001 |
| Han | 4902(37.5%) | 766(54.8%) |  |
| Uygur | 4588(35.1%) | 128(9.2%) |  |
| Kazak | 3591(27.4) | 503(36%) |  |
| Smoking | 3759(28.7%) | 388(27.8) | 0.499 |
| Hypention | 4906(37.5%) | 880(63%) | ＜0.001 |
| Diabetes | 756(5.8%) | 109(7.8%) | 0.002 |
| glucose | 5.12±1.66 | 5.28±1.67 | ＜0.001 |
| Dyslipidemia | 6487(49.6%) | 678(48.5%) | 0.452 |
| TG | 1.57±1.28 | 1.41±1.05 | ＜0.001 |
| TC | 4.59±1.13 | 4.7±1.17 | ＜0.001 |
| LDL-C | 2.88±0.92 | 2.84±0.90 | 0.104 |
| HDL-C | 1.27±0.45 | 1.26±044 | 0.712 |
